# TOPical Imiquimod treatment of high-grade Cervical intraepithelial neoplasia (TOPIC trial): study protocol for a randomized controlled trial

**DOI:** 10.1186/s12885-016-2187-3

**Published:** 2016-02-20

**Authors:** M. M. Koeneman, A. J. Kruse, L. F. S. Kooreman, A. zur Hausen, A. H. N. Hopman, S. J. S. Sep, T. Van Gorp, B. F. M. Slangen, H. J. van Beekhuizen, M. van de Sande, C. G. Gerestein, H. W. Nijman, R. F. P. M. Kruitwagen

**Affiliations:** Department of Obstetrics and Gynaecology, Maastricht University Medical Center, Post box 5800, 6202 AZ Maastricht, The Netherlands; GROW, School for Oncology and Developmental Biology, Maastricht University Medical Center, Maastricht, The Netherlands; Department of Pathology, Maastricht University Medical Center, Maastricht, The Netherlands; Department of Molecular Cell Biology, Maastricht University Medical Center, Maastricht, The Netherlands; Department of Internal Medicine, Maastricht University Medical Center, Maastricht, The Netherlands; Department of Obstetrics and Gynaecology, Erasmus MC Cancer Institute, Rotterdam, The Netherlands; Department of Obstetrics and Gynaecology, Meander Medical Center, Amersfoort, The Netherlands; Department of Obstetrics and Gynaecology, University Medical Center Groningen, Groningen, The Netherlands

**Keywords:** Cervical intraepithelial neoplasia, Imiquimod, Biological markers, Human papillomavirus, Natural history

## Abstract

**Background:**

Cervical intraepithelial neoplasia (CIN) is the premalignant condition of cervical cancer. Whereas not all high grade CIN lesions progress to cervical cancer, the natural history and risk of progression of individual lesions remain unpredictable. Therefore, high-grade CIN is currently treated by surgical excision: large loop excision of the transformation zone (LLETZ). This procedure has potential complications, such as acute haemorrhage, prolonged bleeding, infection and preterm birth in subsequent pregnancies. These complications could be prevented by development of a non-invasive treatment modality, such as topical imiquimod treatment.

The primary study objective is to investigate the efficacy of topical imiquimod 5 % cream for the treatment of high-grade CIN and to develop a biomarker profile to predict clinical response to imiquimod treatment. Secondary study objectives are to assess treatment side-effects, disease recurrence and quality of life during and after different treatment modalities.

**Methods/design:**

The study design is a randomized controlled trial. One hundred forty women with a histological diagnosis of high-grade CIN (CIN 2–3) will be randomized into two arms: imiquimod treatment during 16 weeks (experimental arm) or immediate LLETZ (standard care arm). Treatment efficacy will be evaluated by colposcopy with diagnostic biopsies at 20 weeks for the experimental arm. Successful imiquimod treatment is defined as regression to CIN 1 or less, successful LLETZ treatment is defined as PAP 1 after 6 months. Disease recurrence will be evaluated by cytology at 6, 12 and 24 months after treatment. Side-effects will be evaluated using a standardized report form. Quality of life will be evaluated using validated questionnaires at baseline, 20 weeks and 1 year after treatment. Biomarkers, reflecting both host and viral factors in the pathophysiology of CIN, will be tested at baseline with the aim of developing a predictive biomarker profile for the clinical response to imiquimod treatment.

**Discussion:**

Treatment of high-grade CIN lesions with imiquimod in a selected patient population may diminish complications as a result of surgical intervention. More knowledge on treatment efficacy, side effects and long-term recurrence rates after treatment is necessary.

**Trial registration:**

EU Clinical Trials Register EU-CTR2013-001260-34. Registered 18 March 2013.

Medical Ethical Committee approval number: NL44336.068.13 (Medical Ethical Committee Maastricht University Hospital, University of Maastricht).

Affiliation: Maastricht University Hospital.

Registration number ClinicalTrials.gov: NCT02329171.

## Background

Cervical intraepithelial neoplasia (CIN) is the premalignant condition of cervical cancer and is caused by cervical human papillomavirus (HPV) infection [1]. The natural history of individual CIN lesions is unpredictable. Approximately 30 % of high-grade CIN progresses to cervical cancer [2, 3]. On the contrary, recent evidence suggests that spontaneous regression occurs in approximately 20–40 % of high-grade lesions [4–7]. Current histopathological assessment is unable to differentiate between lesions that will progress to cervical cancer and those that will regress spontaneously. Therefore, all high-grade CIN lesions are currently treated by surgical excision, consisting of large loop excision of the transformation zone (LLETZ). This treatment is associated with potential complications. Short term complications include pain, vaginal discharge and bleeding. The most serious late complication is premature birth in subsequent pregnancy, probably due to cervical insufficiency [8–10]. Evidence shows a two fold increase in premature birth between 32/34 and 37 weeks in patients who were treated with LLETZ. Furthermore, cervical surgery for CIN may be associated with subfertility. A recent case–control study in 152 patients who underwent cervical surgery showed a twofold increase in prolonged time to conception (16.4 % vs 8.6 %) for patients who underwent LLETZ [11]. Since cervical dysplasia is most common in women of childbearing age, these potential complications are of special interest to the patient population and surgical intervention should be avoided if possible. Therefore, an effective non-invasive treatment modality is needed. A potential agent in non-invasive therapy is imiquimod cream. Imiquimod is an immunomodulator with antiviral and anti-tumour effects. It is a toll-like receptor 7 agonist and induces up regulation of interferon and activation of dendritic cells [12]. It is currently used in treatment of basal cell carcinoma, actinic keratosis and external genital warts in adults. As an off-label drug, it has also been proven effective in the treatment of HPV related vulvar intraepithelial neoplasia (VIN) [13]. The use of imiquimod in CIN has been studied by several authors [7, 14, 15]. Only one randomized controlled trial was conducted, evaluating the efficacy of imiquimod treatment in high-grade CIN [7]. Grimm et al. included 59 patients, who were randomized for treatment with imiquimod or placebo during 16 weeks. The study results are promising: both histologic regression and complete remission of high-grade CIN was significantly more frequent in patients treated with imiquimod, compared to the control arm (73 % vs 39 % and 47 % vs 14 % respectively). However, side-effects seem common and long-term outcomes are unknown.

Ideally, the response to imiquimod treatment of an individual patient would be predictable. Previous studies have shown that the natural behaviour of CIN lesions can be partially predicted by biomarker models, consisting of markers that reflect host, viral and cellular factors [4, 16]. This study aims to develop a similar biomarker prediction model for the individual response to imiquimod treatment, in order to enable individualized treatment of CIN lesions.

This study aims to confirm the short and long term efficacy of imiquimod 5 % cream in the treatment of high-grade CIN, as well as to evaluate clinical applicability by assessment of side-effects and quality of life during and after treatment. Additionally, it aims to develop a biomarker prediction model for clinical response to imiquimod treatment of high-grade CIN. For this purpose, we designed a randomized controlled trial with two arms, in which imiquimod treatment is compared to standard treatment by LLETZ. The trial was designed according to the CONSORT guidelines.

## Methods

### Setting and study population

The study will be started at the outpatient clinic of gynecologic oncology, Maastricht University Medical Center, the Netherlands and is intended as a multi-center trial in the future. Inclusion criteria are newly diagnosed, histologically confirmed high-grade CIN lesions (CIN 2–3) and age above 18 years. Exclusion criteria are previous histologically confirmed high-grade CIN (CIN 2–3), concomitant vulvar and/or vaginal intraepithelial neoplasia, previous cervical malignancy, current malignant disease, immunodeficiency (including HIV/AIDS and immunodepressive medication), pregnancy or lactation and legal incapability.

### Study objectives and outcome measures

The primary study objectives and outcome measures are:

1. Assessment of treatment efficacy of imiquimod treatment for high-grade CIN, as compared to LLETZ treatment. Successful treatment for the experimental (imiquimod) arm is defined as regression to CIN 1 or less in diagnostic biopsies at 20 weeks follow-up. Successful treatment for the LLETZ arm is defined as normal cytology at 6 months follow-up. Based upon earlier studies, we hypothesize that 50–75 % of patients in the experimental (imiquimod) arm will show regression to CIN 1 or less.
  These two different outcome measures (cytology in the LLETZ arm and histology in the experimental arm) were selected in order to optimize the assessment of treatment efficacy, whilst limiting overtreatment of patients (performing unnecessary biopsies or LLETZ treatments). For the experimental arm, it is assessed by colposcopy with diagnostic biopsies and for the LLETZ arm it is assessed by cytology. Regarding efficacy of LLETZ treatment, the significance of resection margins of the LLETZ specimen is controversial and is not advised in guidelines as outcome measure of LLETZ efficacy. Therefore, regular follow-up cytology at six months was selected as outcome measure, as is also done in clinical practice. Regarding imiquimod treatment, histological assessment of treatment efficacy was selected as outcome measure, in order to optimize the assessment of potential residual disease. Biopsies were chosen rather than a standard LLETZ procedure to evaluate residual disease, to prevent overtreatment.
2. Development of a biomarker model to predict adequate response to imiquimod treatment.

Secondary study objectives and outcome measures are:

1. To determine the incidence and severity of side effects of LLETZ and imiquimod therapy, scored by the Common Terminology Criteria for Adverse Events guidelines.
2. To estimate disease recurrence rates for both arms at 6, 12 and 24 months follow-up, defined as abnormal cervical cytology. The follow-up term starts after treatment is finished.
3. To assess Quality of life (QoL) for both arms before, during and after treatment (at 0 and 20 weeks and after 1 year) by the following QoL questionnaires: [1] Medical Outcomes Study 36-Item Short-Form General Health Survey (RAND 36), to assess generic health-related quality of life, [2] the European Organization for Research and Treatment of Cancer (EORTC) QLQ-C30, to assess cancer-specific health-related quality of life, and [3] the European Organization for Research and Treatment of Cancer (EORTC) QLQ-CX24, to assess cervical cancer specific quality of life, including sexual functioning.

### Interventions

After informed consent is obtained, patients are equally randomized into one of two arms:

1. *Experimental arm*. Patients in this arm are treated by a 16-week regime of imiquimod 5 % cream.
2. *Standard arm*. Patients in this arm receive standard treatment by LLETZ.

Follow-up visits are anchored to the start of the treatment (either imiquimod or LLETZ). Cytological assessment will be performed by two independent trained cytology analysts, according to the Papanicolaou system. In case of inconsistent results, a consensus will be reached by discussion. The histopathological assessment of cervical biopsies will be performed by two independent pathologists according to national guidelines, based on the WHO guidelines. In case of inconsistent results, a consensus will be reached by discussion. CIN diagnosis will be based on evaluation of histological features concerned with differentiation, maturation and stratification of cells and nuclear abnormalities, in combination with p16 staining. In CIN 1 there is good maturation with minimal nuclear abnormalities and few mitotic figures. Undifferentiated cells are confined to the deeper layers (lower third) of the epithelium. Mitotic figures are present, but not very numerous. Cytopathic changes due to HPV infection may be observed in the full thickness of the epithelium. CIN 2 is characterized by dysplastic cellular changes mostly restricted to the lower half or the lower two-thirds of the epithelium, with more marked nuclear abnormalities than in CIN 1. Mitotic figures may be seen throughout the lower half of the epithelium. In CIN 3, differentiation and stratification may be totally absent or present only in the superficial quarter of the epithelium with numerous mitotic figures. Nuclear abnormalities extend throughout the thickness of the epithelium. Many mitotic figures have abnormal forms.

Patients in the experimental arm are treated with imiquimod 5 % cream during 16 weeks. Imiquimod 5 % cream is administered in a vaginal applicator, containing 12,5 mg of imiquimod (one sachet). The cream is administered three times per week. The cream is administered by patients themselves at night, before going to bed. A vaginal shower is performed in the morning in order to remove cream remainders. In case of mild systemic drug-related side effects, patients are offered a prescription for anti-inflammatory drugs (paracetamol or NSAID). In case of more severe or persistent systemic or severe local side effects (Common Terminology Criteria for Adverse Events grade 2 or higher), the frequency of imiquimod application is decreased to twice per week, and subsequently to once per week if side effects persist. Imiquimod treatment is discontinued if side effects are unacceptable to patients hereafter. Subjects should use adequate contraception in order to prevent pregnancy. Subjects should not have vaginal sexual intercourse from the time of application of the imiquimod cream until the vaginal shower the next morning. A control colposcopy with diagnostic biopsies is performed after 10 weeks to rule out disease progression. Biopsies are performed at the initial high-grade CIN lesion site and at any other suspect site, with a minimum of two. In case of progressive disease (defined as increase in lesions size with stable disease grade, higher disease grade or invasive disease), surgical excision is performed.

Treatment efficacy for the experimental arm is evaluated at 20 weeks follow-up, by colposcopy with diagnostic biopsies. Biopsies are performed by using a cervical biopsy specimen forceps (Aesculap ER055R), at the initial high-grade CIN lesion site and at any other suspect site, with a minimum of two. In case of persistent or progressive disease (> CIN 1), surgical excision is performed.

**Fig. 1:**
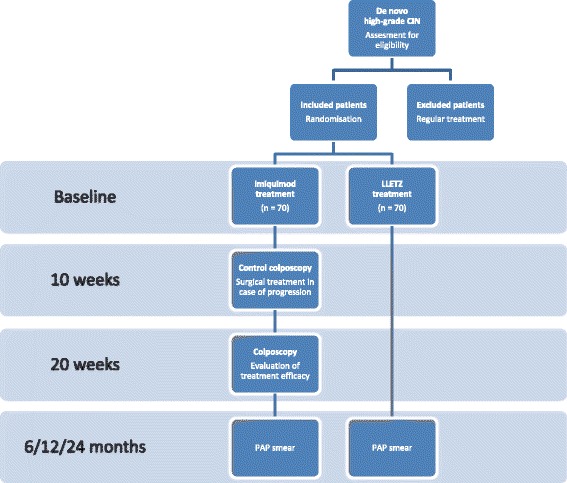
Summary of study interventions

Patients in the standard treatment arm undergo LLETZ at short term (within 4 weeks after the diagnosis). Excision of the transformation zone and macroscopic lesions is performed by a monopolar loop electrode, under local anaesthesia. A summary of study interventions can be found in Fig. 1.

The imiquimod treatment period was set at 20 weeks, in order to realize adequate treatment efficacy of imiquimod, while minimizing the risk of progression of cervical dysplasia to invasive disease. Progression of CIN into cervical cancer is considered to be a slow process. The annual risk of progression of CIN 3 to invasive cervical cancer is estimated to be less than 1 % [17]. We identified ten studies in which patients with high-grade CIN underwent watchful waiting for a set period of time [4, 6, 7, 18–24]. A total of 637 patients were included either as a control group, receiving no treatment during 6 weeks to 15 months, or followed during the period between diagnosis and LLETZ. Three cases of invasive disease were identified: all occurred in the same study after 16 weeks of observation. The possibility of invasive disease already present at the initial colposcopy (due to biopsy error) cannot be excluded. Based on these results, we set the maximum treatment period at 20 weeks and we included an additional control colposcopy with diagnostic biopsies after 10 weeks. When no disease progression is detected during this colposcopy, the imiquimod treatment can be continued until the 20 weeks colposcopy.

### Sample size calculation

The sample size calculation was based on a regression rate of 73 % at 20 weeks follow-up after immunotherapy and a 95 % treatment efficacy of LLETZ. Using 80 % power and alpha = 5 %, the estimated sample size required is 42 women in both arms. Allowing for a withdrawal rate of approximately 20 %, 53 women will have to be recruited in each arm. Sample size calculations for the secondary outcome measures indicated that roughly 70 patients per treatment arm would be necessary. Although the sample size calculation should be based on the primary outcome measure, we decided to recruit a total of 140 patients (70 per arm).

### Randomization

Randomization is performed by the principal investigator to prevent selection and allocation bias, by use of a computerized randomization tool. Sampling is stratified according to the following age categories: 18–24, 25–39, and 40 years and older. Sampling will be stratified according to study centre.

### Data collection

Coded data are stored both on paper and in an electronic database. Collected data are stored in a digital case report form (CRF). Raw data is available only to the principal and coordinating investigator. The following data are recorded:

*Baseline*

- Patients characteristics: age, ethnical background, education, medical history, smoking, sexual behaviour.
- HPV genotyping.
- Histological biomarkers on biopsies of patients in the experimental arm: markers of lymphoproliferative response: CD4, CD8, CD25, CD138, fox p3; cell cycle markers: p16, Rb, p53, Ki67, CK 13, CK 14, IMP3.
- Quality of life.

*6 weeks follow-up*

- Adverse effects of imiquimod treatment: patient reported side effects and side effects noticed at clinical investigation.
- Adverse effects of LLETZ treatment: patient reported side effects, using a standardized report form.

*10 weeks follow-up*

- Treatment compliance: amount of applied doses of imiquimod, as documented by the study subject on a dose calendar.
- Histological presence and grading of CIN for the experimental arm.
- Adverse effects of imiquimod treatment: patients reported side effects and side effects noticed at clinical investigation.

*14 weeks follow-up*

- Adverse effects of imiquimod treatment: patients reported side effects and side effects noticed at clinical investigation.

*20 weeks follow-up*

- Treatment compliance: amount of applied doses of imiquimod, as documented by the study subject on a dose calendar.
- Histological presence and grading of CIN for the experimental arm.
- Adverse effects of imiquimod treatment: patients reported side effects and side effects noticed at clinical investigation.
- Quality of life for all patients.

*6, 12, 24 months follow-up*

- Cervical cytology outcomes for all patients, including HPV genotyping.
- Quality of life for all patients at 12 months follow-up

### Statistical methods

Logistic regression analysis will be used to evaluate treatment efficacy of imiquimod treatment, compared to LLETZ treatment. Covariates in this analysis are age at diagnosis, CIN grade, smoking and HPV-subtype. Analysis will be based on intention-to-treat protocol. A biomarker prediction model for adequate response to imiquimod treatment will be developed by backward logistic regression analysis of biomarkers based on likelihood ration tests. The prevalence and severity of side effects of imiquimod and LLETZ treatment will be presented as proportions and means with 95 % confidence intervals. Differences in the rates of overall side-effects and severe side-effects between the imiquimod and LLETZ arms will be tested with a chi-square test. Disease recurrence rates after 6, 12 and 24 months will be evaluated in successfully treated patients by use of multiple logistic regression analysis, after adjustment for age at diagnosis, CIN grade, smoking, sexual behaviour and HPV subtype. Repeated-measures analysis of variance will be used to test for between-group differences over time in Quality of Life scores. Analysis of covariance will be used to compare group scores on these outcomes at 20 weeks and at 12 months, with adjustment for baseline scores.

### Withdrawal of individual subjects and replacement

Subjects can leave the study at any time for any reason, without consequences. The investigator can decide to withdraw a subject from the study for medical reasons. Withdrawn individuals are not replaced by a new volunteer. Patients who withdraw from the study are offered the standard treatment of CIN, being a LLETZ procedure with standard follow up.

### Ethical considerations and dissemination

The study was approved off by the Medical Ethical Committee of Maastricht University Hospital, University of Maastricht. The study will be performed according to the standards outlined in the Declaration of Helsinki. Ethics committee approval has been completed. Monitoring of the study is performed by a Data and Safety Monitoring Board, appointed by the Clinical Trial Center Maastricht. Adverse events are recorded and reported according to local protocol. Study results will be offered for publication in an international medical journal. Study results will be communicated to trial participants by mail, if agreed upon by the participant.

### Substantial amendment

The current study protocol includes a substantial amendment to the original study protocol, which consisted of three study arms: imiquimod treatment arm, LLETZ treatment arm and an observational arm. The purpose of the observational arm was to assess spontaneous regression of high-grade CIN and to develop a prognostic biomarker panel to predict spontaneous regression of high-grade CIN. Patients in the observational arm underwent no treatment for a period of maximum 20 weeks. Histological assessment of disease development was performed after 10 and 20 weeks by colposcopy with diagnostic biopsies. Inclusion of patients into the study was hampered by the observational arm: patients declined the study because they wished to be treated, rather than undergo observational management. The observational arm was removed from the study.

## Discussion

The development of a non-invasive treatment modality for high-grade CIN lesions may diminish complications as a result of surgical intervention. An earlier study indicates that imiquimod induces disease regression in 73 % [7, 13]. Thus, imiquimod treatment may prevent surgical treatment in the majority of patients. The current study aims to test the treatment efficacy hypothesis as well as long term disease recurrence after treatment and clinical applicability of imiquimod treatment, defined as side effects and quality of life during and after treatment. Furthermore, it aims to develop a prediction model for clinical response to imiquimod treatment, based on histological biomarkers.

### Trial status

First approved by the Medical Ethical Committee Maastricht University 1Hospital, University of Maastricht, on 21 May 2014. Recruitment started in December 2014. Protocol version 5.0.

## Abbreviations

CIN: cervical intraepithelial neoplasia
LLETZ: large loop excision of the transformation zone
HPV: human papillomavirus
VIN: vulvar intraepithelial neoplasia
